# Clinical and radiological outcomes of open reduction alone versus open reduction with pelvic osteotomy for developmental dysplasia of the hip in children over 1.5 years of age

**DOI:** 10.25122/jml-2023-0212

**Published:** 2023-12

**Authors:** Ali Saleh Aljanabi, Wissam Saleh Hakim

**Affiliations:** 1 Department of Surgery, College of Medicine, University of Al-Qadisiyah, Al Diwaniyah, Iraq

**Keywords:** femoral osteotomy, unilateral developmental dysplasia, Pemberton osteotomy, surface electromyography

## Abstract

Developmental dysplasia of the hip (DDH) is commonly addressed through surgical intervention, usually performed in a specialized tertiary care facility. The purpose of this study was to evaluate the surgical outcomes in patients with DDH who had open reduction alone or in conjunction with bone surgery at our facility. We retrospectively reviewed the medical records of patients with DDH, categorizing them into two groups: Group OR underwent open reduction (OR) alone, and group ORBO underwent OR in conjunction with femoral or pelvic osteotomies. The modified McKay classification was used to evaluate clinical outcomes, and the Severin classification was used to evaluate radiological outcomes. Avascular necrosis and other postoperative issues were observed. Our cohort consisted of 66 patients (76 hips), with a mean age at surgery of 1.8±2.6 and a follow-up period ranging from one to three years. Clinically, 48 out of 66 patients achieved satisfactory outcomes, and radiologically, 47 patients were classified as satisfactory. Although there was no statistically significant difference in the radiological outcome (P=0.85), more patients in the OR group than in the ORBO group (P=0.05) had better outcomes. Avascular necrosis (AVN) was observed in 23 hips (34.8%), with Grade I AVN being the most prevalent in 19 hips that underwent OR with bone surgery (63.2%). The occurrence of AVN was associated with poorer clinical and radiological outcomes (P=0.05). Overall, the DDH operation at our center had positive outcomes. The OR group showed better clinical outcomes despite similar radiological findings and AVN rates compared to the OR with bone surgery group. The presence of AVN was linked to poor clinical and radiological outcomes.

## INTRODUCTION

Early detection makes developmental dysplasia of the hip (DDH) easier to treat. The extraordinary remodeling capacity of the acetabulum in the first year of life is demonstrated by the high success rate of therapy with the Pavlik bandage. Due to the high success rate of early treatment choices and the low complication rates, early screening procedures to diagnose DDH have been used in a number of countries [1]. Although there is still much disagreement about the efficacy of universal screening, some evidence points to the failure of screening and the initiation of treatment as reasons for an indication of open reduction [1]. Patients requiring open-reduction surgery typically present earlier for treatment compared to those undergoing closed reductions or splinting [1]. The criteria for defining a 'late-onset' patient with DDH lacks precision. A recent Australian study considered that detection of DDH after three months of age was associated with 'late diagnosis'. Although some studies suggest that the acetabular index may continue to normalize up to eight years of age if the hip is concentrically reduced, data beyond 12 to 14 months are scarce [2]. Studies suggest that the reduction treatments performed during this time significantly slow acetabular normalization. A child ten months and older is 12 times more likely to need open reduction than a child diagnosed before six weeks of age [2].

Acetabular dysplasia, characterized by a shallow or abnormally oriented acetabulum, predisposes to accelerated arthritis progression due to abnormal edge loading and insufficient coverage to maintain the reduced position of the femoral head [2]. Persistent dysplasia unresponsive to initial treatments may necessitate pelvic osteotomies, which facilitate more normal acetabular development [2]. The hip socket has been documented to remodel over a period extending up to five years, with continuous improvement observed when the hip is positioned correctly [3]. Therefore, these procedures are often only performed on older children. As a result, the timing of the osteotomy is debatable, though it is commonly performed between the ages of three and five in cases of residual acetabular dysplasia [3].

The Salter, Pemberton, and Dega osteotomies are the three most prevalent procedures, differing primarily in the direction and extent of the osteotomy above the acetabulum [3]. Current evidence suggests that the clinical and radiological outcomes of these various osteotomy techniques for treating residual acetabular dysplasia are comparably effective [3]. Although there is no absolute age contraindication for these osteotomies, achieving sufficient acetabular coverage in older children with a single osteotomy can be challenging [3]. A triple innominate osteotomy may be an option for older children, often >6 years old, with an open tri-radiate cartilage development center. In the triple innominate osteotomy, all three osseous zones around the acetabulum are sliced, allowing for the free reorientation of the acetabulum and greater acetabular dysplasia correction [3].

The aim of this study was to evaluate and compare the clinical and radiological outcomes of open reduction alone versus open reduction combined with pelvic osteotomy in the treatment of developmental dysplasia of the hip in children over 1.5 years of age.

## MATERIAL AND METHODS

We conducted a retrospective review of the medical records of patients treated surgically for DDH at Al-Diwaniyah Teaching Hospital from January 2020 to January 2022. The inclusion criteria encompassed all patients with unilateral or bilateral DDH who underwent surgery within this timeframe. Exclusion criteria were patients with severe, septic, paralyzed, or teratologic hip dislocations. All patients involved in the study were monitored for at least two years after their operation.

### Surgical procedures

Patients were stratified into two groups based on the surgical intervention received. Group OR consisted of patients who underwent open reduction (OR) alone, while Group ORBO included those who underwent OR in conjunction with femoral or pelvic osteotomies. This study used a modified Smith-Petersen approach for anterior access with bikini line skin incisions to perform open reductions. The Klisic method, which measures femoral shortening, was used when there was enough soft tissue tension to prevent shortening. For patients requiring additional corrective measures post-hip reduction, pelvic osteotomies were conducted using the Salter and Pemberton techniques. Both patient groups of young individuals underwent three months of postoperative hip spica casts. The treatment was carried out by skilled pediatric orthopedic surgeons (SI and AHR).

### Outcome measures and data collection

A review of recorded clinical data and follow-up radiographs allowed us to assess outcome measures. Clinical outcomes for each group were calculated using the modified McKay grading. McKay Grades I and II are defined as 'excellent' and 'good' respectively, representing satisfactory outcomes, while Grades III and IV are considered 'fair' and 'poor', reflecting unsatisfactory outcomes. Radiological results were assessed using the classification according to Severin *et al*., with classes I and II rated as 'satisfactory' and classes III and IV as 'unsatisfactory'. A review of medical records revealed difficulties during and after surgery. Avascular necrosis (AVN) of the hip, when present, was classified according to the Bucholz and Ogden system [8]. One investigator was solely responsible for data collection. For analysis, data were specifically collected on the more severely affected hips in cases of bilateral DDH to ensure consistency in assessing the outcomes of surgical interventions.

### Statistical analysis

The data collected were analyzed using SPSS software (version 25.0). Categorical data were evaluated using the Chi-square test and Fisher’s exact test where applicable. A p-value threshold of 0.05 was used to determine statistical significance.

## RESULTS

The records of 66 patients (76 hips) were reviewed, with an average age of 1.8±2.6 years at the time of surgery and 1.9±2.8 years at the most recent follow-up. The follow-up period ranged from one to three years. The sample consisted of nine male patients (13.6%) and 57 female patients (86.4%) (Table 1). Children in the ORBO group (open reduction with bone osteotomy) were significantly older than those in the OR group.

**Table 1:** Patient demographics and characteristics

	OR (n=16) Mean ± SD	ORBO (n=50) Mean ± S.D	P value
Age at surgery (years)	1.8±2.6	2.5±1.5	<0.05
Age at last follow-up (years)	1.9±2.8	3.0±1.4	<0.05
Gender (Male/Female)	6/21	3/36	0.49*
Laterality			
Left	7	28	0.55*
Right	7	14	
Bilateral	2	8	

**Figure 1:**
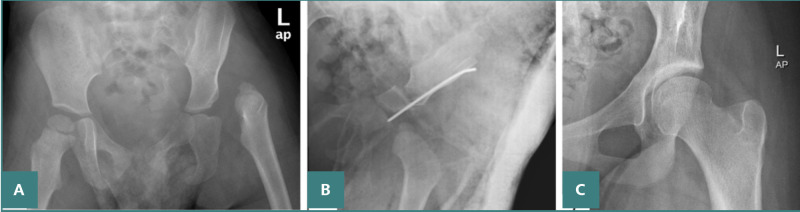
Post-operative X-ray of a patient following open reduction. A: Salter osteotomy, B-C: Anteroposterior left hip X-ray at the age of 11.

**Figure 2:**
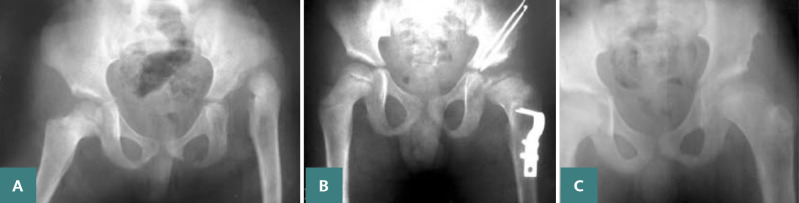
Post-operative X-ray of a patient who underwent open reduction, femoral shortening, and Salter osteotomy. A: Salter osteotomy, B: Post-operative X-ray showing a retroverted hip; Salter osteotomy with femoral graft. Removal of K-wire from bone transplant. C: X-ray outcome at 6 years classified as Severin class III.

Clinical results using modified McKay grading at the final follow-up revealed that 48 patients obtained grades I (excellent), II (good), and III (poor). There was no grade IV patient (poor). According to radiological data, Severin classification revealed that 47 patients were class I, 12 were class II, and seven were class III. AVN was observed in 23 hips (34.8%), predominantly grades I (21.2%), II (10.6%), and III (3%). Figure 1 depicts a case study of a patient who received satisfactory results, while Figure 2 depicts a case study of a patient with unsatisfactory outcomes.

The chi-square test demonstrated that the proportion of unsatisfactory results in the OR group was substantially lower (P<0.05) compared to the ORBO group (P=0.80) (Table 2). No difference was observed in AVN occurrence between the groups (χ^2^=2.66, P=0.63). However, the presence of AVN was associated with increased unsatisfactory clinical and radiological outcomes (P<0.05) (Table 3).

**Table 2:** Clinical outcomes by McKay grading and Severin Class

Group	McKay grading		Total	2	P value
	Satisfactory	Unsatisfactory		4.71	<0.05*
OR	15	1	16		
ORBO	33	17	50		
Total	48	18	66		
	**Severin class**				
	Satisfactory	Unsatisfactory		0.06	0.80
OR	11	5	16		
ORBO	36	14	50		
Total	47	19	66		

**Table 3:** Correlation between AVN and modified McKay grading

AVN	McKay grading		Total	2	P value
	Satisfactory	Unsatisfactory		8.32	<0.05*
No AVN	36	8	43		
Grade 1	10	9	19		
Grade 2	2	0	2		
Grade 3	0	1	1		
Total	48	18	66		
**Severin class**					
	Satisfactory	Unsatisfactory	Total	1	<0.05*
No AVN	36	7	43		
Grade 1	6	8	14		
Grade 2	3	4	7		
Grade 3	0	2	2		
Total	45	21	66		

We also looked at whether the patient's age at the time of the initial surgery had an impact on the outcomes. There was no difference in McKay grading (P=0.34), Severin class (P=0.23), or incidence of AVN (P=0.28) between patients who had surgery before and after reaching three years of age.

Before the last follow-up, seven patients (eight hips) had undergone multiple surgeries. These patients underwent either open reduction with additional femoral or pelvic surgery or repeated open reduction of the hip. This subgroup also included cases of subluxated hips (five hips) or dislocated hips (three hips) within the cohort. Among the remaining nine patients (16 hips) who had already undergone closed reductions (with laxity) prior to their index treatment, there were no significant differences in McKay grading (P=0.29), Severin grade (P=0.38), or the incidence of AVN (P=0.14) compared to those who received only one therapy.

## DISCUSSION

According to our findings, the majority of patients who underwent open surgery, whether or not they also underwent femoral/pelvic surgery, experienced positive clinical (75.8%) and radiological (72.7%) outcomes over the course of the procedure.

The term 'satisfactory' in our context referred to outcomes classified as 'excellent' or 'good' based on Severin I and II radiological classes as well as McKay's clinical grade. This aligns with numerous publications reporting positive outcomes ranging from 70% to 90% in studies utilizing various DDH procedures with varying follow-up durations [9]. El-Sayed [10] reported favorable outcomes from a one-stage procedure combining open reduction with femoral and pelvic osteotomies, noting an 80–90% rate of satisfactory medium-term results over 3-6 years. Our findings are consistent with these reports, underscoring the effectiveness of surgical intervention in DDH management. Bhuyan *et al*. observed that 78% of patients who reached skeletal maturity over an average of 18 years had satisfactory radiological outcomes [11].

Nearly 80% of children who underwent Pemberton reported excellent clinical and radiological outcomes [12]. In our study, children who received only open reduction had superior clinical outcomes compared to those who underwent open reduction with extra bone surgery. This is in line with more recent thorough analyses that discovered that the OR group was more likely to experience positive clinical and radiological outcomes. Our study, however, did not identify any variations in radiologic outcomes between groups. Despite having positive outcomes, the OR group had an unacceptable high reoperation rate, and they were up to 10-15 times more likely than the ORBO group to need non-salvage intervention, according to the same thorough research [13].

The OR group outperformed the ORBO group, which may be explained by the OR group's younger age at surgery. Age discrepancies between OR groups are unavoidable because ORBO therapy is often advised for kids ages 18 months and older. Infants in the OR group under 18 months in a Turkish research had lower rates of AVN and reoperation rates than infants in the ORBO group over 18 months [14]. The outcomes of both the clinical and radiological exams were identical. Children who had surgery after the age of three had worse radiological outcomes than adults, according to research by Holman *et al*. [15]. Many of our patients were still developing their skeletons at their last follow-up assessment, but our study could not show this age effect. Ning *et al*. conducted a study focusing on index tampering and categorized patients into three age groups: 1.5–2.5 years, 2.5–8 years, and over 8 years. Their comprehensive analysis, which included over 800 hips, demonstrated that one-stage surgeries conducted on patients aged between 2.5 and 8 years yielded the most favorable radiological outcomes, together with the lowest incidence of AVN rates [16].

One of the limitations of our study was the single-center design and the small cohort of patients. We recommend larger, multicentre studies to further investigate DDH treatment. Given the prevalence of DDH and the potential for excellent prognosis with early and appropriate intervention, extensive research is crucial for refining treatment strategies and optimizing patient outcomes.

## CONCLUSION

The outcomes of DDH surgery performed at our institution were comparable to those from other studies. Radiological results and AVN rates were similar in the OR and ORBO groups, although the OR group achieved better clinical outcomes. If left untreated at a young age, it can lead to gait disturbance, limited hip movement, joint pain, and osteoarthritis. However, the optimal timing for surgical intervention remains a subject of debate, primarily due to the increased risk of complications such as AVN and inadequate hip development associated with surgery in older children. These challenges are exacerbated in cases where deformities of the acetabulum or femoral head are present or when soft tissue contractures place undue pressure on the femoral head during reduction attempts. The intricate femoral osteotomy surgery can be safely carried out in a single phase. This involves shortening and counter-rotation osteotomies, as well as pelvic osteotomy. This one-step operation is the preferred treatment for congenital hip dislocation, which has only recently been identified in older children. This one-step surgical strategy aims to mitigate the risks of AVN, joint stiffness, and functional impairment, optimizing long-term hip function and patient quality of life by addressing the critical issue of femoral head pressure.
